# Supplementary material for: Are carbon emissions trading and green financial instruments synergistic? -Comprehensive quantitative research based on content analysis
Source: PLoS One. 2024 Mar 7;19(3):e0298601. doi: 10.1371/journal.pone.0298601 (PMC10919601; doi:10.1371/journal.pone.0298601)
Supplement: S1 Data — (ZIP) [file pone.0298601.s001.zip › Data/地级市Z_F工作报告.zip!/╣π╢1⁄2/╓╨╔╜╩╨/└┤╫╘╣1⁄2╓┌║┼í╛┬φ┐╦╩2╛▌═°í┐_2018.docx]

各位代表：

　　我代表市人民政府向大会作政府工作报告，请予审议，并请政协委员和其他列席人员提出意见。

■2017年工作回顾

　　2017年是全市上下凝心聚力、砥砺奋进、取得重要成绩的一年。一年来，市政府在省委、省政府和市委的正确领导下，在市人大及其常委会和市政协的监督支持下，认真学习贯彻党的十九大精神，深入贯彻落实习近平总书记对广东作出的“三个定位、两个率先”和“四个坚持、三个支撑、两个走在前列”重要指示批示精神，坚持稳中求进工作总基调，深入推进供给侧结构性改革，大力实施创新驱动发展战略，着力构筑新型比较优势，全市经济运行稳中向好，社会大局和谐稳定，城市竞争力不断提高。

　　（一）经济发展更加稳健。适应把握经济发展新常态，打出一整套提质增效组合拳，经济发展质量和效益明显提高。预计实现地区生产总值3500亿元左右，人均生产总值超过10万元大关，地均生产总值每亩约12万元，均处全省前列。中国社会科学院与联合国人居署共同发布的全球城市竞争力报告显示，中山可持续竞争力排珠三角第4，经济竞争力排珠三角第5。经济结构更加趋好，先进制造业增加值占规上工业增加值比重提高至44.6%，重工业增加值占规上工业增加值比重提高至41.6%，现代服务业增加值占服务业增加值比重提高至61.3%。招商引资成效突出，4个百亿级项目签约，招商项目总数、投资额、动工数分别增长274%、115%、137%。投资的关键性作用有效发挥，省市重点项目均超额完成任务。市场主体更加活跃，新增各类市场主体7.3万户，增长21.4%。金融支撑更加有力，全市上市上板企业102家。农业现代化稳步推进，建设高标准基本农田3.6万亩，我市出口到澳门的蔬菜占当地市场的50%，出口到香港、澳门的四大家鱼分别占当地市场的40%和70%，粮食安全得到保障。

　　（二）创新动力更加强劲。把创新驱动发展作为核心战略，围绕建设区域科技创新研发中心，实施一揽子政策措施，创新综合能力持续提升。预计研发经费支出占生产总值比重达2.4%，居全省前列。有效发明专利拥有量 5456 件，增长35.2%。珠三角国家自主创新示范区建设加快推进，火炬开发区获批国家“双创”示范基地，翠亨新区获批省级“双创”区域示范基地，成功承办2017年广东省“双创”活动周主会场活动。创新平台建设加快推进，以色列创新中心、中国科学院大学创新中心等高端研发平台落地，哈工大机器人“人工智能创新中心”挂牌，省级以上创新平台增至385家。企业创新能力不断增强，高新技术企业认定数量继续翻番增长，预计超过1700家，实现5亿元以上工业企业研发机构全覆盖。科技金融加速融合，创业投资基金增至45支，资金规模达128亿元，专利质押融资风险共担 “中山模式”向全国推广。人才培育引进力度加大，出台“人才新政18条”，享受国务院政府津贴专家增至39人，纳入国家和省重大人才工程26人，省市创新创业科研团队增至39个，博士后工作平台增至43个，专业技术人才达20.4万人，市技师学院教师梁嘉伟勇夺第44届世界技能大赛信息网络布线项目金牌。中国城市科技创新发展报告（2017年）显示，中山城市科技创新发展指数跻身全国城市前20强、地级市第4。

　　（三）城市建设更加完善。坚持规划先行，土地利用总体规划调整完善方案获省批准。实施组团式发展战略，“一中心、四组团”发展模式有序展开。要素资源统筹能力不断增强，规划建设九大产业平台，引入项目68个，投资额达645亿元。交通基础设施建设全面推进，中山站开通直达北京、上海、桂林、贵阳、南宁、郑州、昆明、长沙等数十个大中城市的高铁服务，南沙港铁路中山段动工建设；深中通道全面开工，中开高速、东部外环高速、西部外环高速动工建设，广中江高速三期、香海高速加快推进，在建高速公路里程达215公里，超过历年所建成高速公路里程的总和；105国道南段、坦洲快线等8条干线公路动工建设，兴教路、轩朗路（二期）通车，长江路、康华路、景观路加快改造，坦洲宝珠南路、汇财街等跨界道路建成通车，打通翠沙路、富康北路、大沙南路等5条断头路；中山港新客运码头开工建设，中山至深圳机场水上客运航线通航。特色小镇加快建设，大涌镇成为国家级特色小镇，东升镇纳入国家运动休闲特色小镇试点，3个特色小镇成为广东省特色小镇创建工作示范点，一批市级特色小镇启动建设。能源、信息等基础设施建设步伐加快，文山输变电工程等一批电力工程加快推进，预计全市光纤入户率达135%。土地集约节约利用水平不断提高，完成 “三旧”改造项目30个，处置闲置土地超万亩。开展市容整治行动，城乡环境综合治理成效显著。

　　（四）改革开放更加深入。认真落实中央和省全面深化改革的部署要求，推动118项重点领域和关键环节改革任务落地。供给侧结构性改革深入推进，105户国有“僵尸企业”全部出清，淘汰印染行业落后产能7600万米，金融机构杠杆率控制在合理水平，通过降低企业税负、减免部分行政事业性收费等综合措施，降低企业各类成本124.2亿元，推进206个补短板项目建设。加大简政放权力度，取消行政许可事项24项，取消行政审批中介服务事项11项，下放火炬开发区、翠亨新区市级经济管理权限144项。以减少审批环节、减少审批资料、减少审批前置条件、减少审批时限为突破，压减行政审批事项申请条件386条、审批环节212个。开展“减证便民”行动，取消145项涉企涉民证明事项。坚持有效市场与有为政府相结合，提高政府服务的精准度和有效性，推出“实体经济十条”“工业用地十条”、公共技术服务一站式对接、企业“管家式”服务等一大批惠企措施，开发惠企导航系统，市镇两级举办112场企业座谈会，帮助企业解决了一大批问题。深化商事制度改革，实施企业登记“多证合一”。“互联网+政务”成效明显，网上办事大厅全流程办理率达98.8%，网上办结率达99.7%，居全省前列。社会信用体系建设取得阶段性成效，建成市公共信用信息管理系统，并在全省率先与网上办事大厅等系统对接，实现信用网上即时核查。深化价格领域改革，下调非居民管道天然气最高限价和居民管道天然气阶梯价格。深化农村综合体制改革，农村土地承包经营权确权登记颁证率达94.7%，完成省下达任务的127%。落实国家“一带一路”战略，经贸拓展活动覆盖半数以上国家和地区。对外贸易成效显著，外贸进出口达2581.5亿元，增长15.4%，新批设立外商直接投资增长104%，实际利用外资增长7.4%。口岸通关环境不断优化，中山国际贸易“单一窗口”标准版上线运行，监管通关信息平台实现中山所有港区全覆盖，企业通关效率大幅提高。成功举办2017粤港澳合作论坛——粤港澳大湾区发展高层峰会、第11届世界中山同乡恳亲大会、中瑞友谊日，对外交流更加频繁。

　　（五）人居环境更加优化。坚决守住生态环保底线，以落实中央环保督察整改要求为抓手，向水污染、大气污染、土壤污染宣战，连续十年在省环境保护责任考核中获优秀等级。营造更多更好的绿色空间，建成翠亨新区翠湖、古镇中心滨河等4个湿地公园和3个组团体育公园，金字山公园、儿童公园、古香林公园、金钟湖公园、气象公园动工建设，完成紫马岭公园儿童设施升级改造，建成龙舟文化主题公园等4个岐江河滨水景观带。城乡绿化美化扎实推进，改造林相3430亩，种植各类树种超14万株。南朗、板芙、古镇、南头成功创建"广东省森林小镇"，数量居全省第1。南区曹边村被国家住建部等多部委联合评为美丽乡村示范村。水污染治理力度持续加大，建立市、镇、村三级河长体系，整治115条内河涌，城区8条黑臭水体完成整治，推进岐江河流域重点区域综合治理。加强大气污染治理，建成紫马岭大气监测超级站，PM 2.5达标，淘汰黄标车4475辆，推广新能源汽车1023辆，建成充电站、充电桩513个，建成全省首个家具行业共性工厂。环保基础设施建设加快推进，中心、北部垃圾处理基地扩容工程动工建设，南部垃圾处理基地投入运营，中心城区723个垃圾屋完成改造，13个农村分散型污水处理项目投入试运行。按照中央环保督察整改要求，清理47家畜禽养殖场和124个饮用水源违法项目，解决了一批环境突出问题。绿色生产生活理念深入人心，入选国家第三批低碳城市试点。

　　（六）民生福祉更加改善。坚持一切为了人民，持之以恒保障和改善民生，一大批惠民举措落地实施，市民获得感不断增强。民生财政支出比重达68.2%。十件民生实事顺利完成。城乡居民收入比排全省前列。就业状况不断改善，城镇新增就业6.1万人，促进创业9515人，中山生源应届毕业生就业率达97.8%，城镇登记失业率为2.3%。社会保障体系不断完善，医保病种分值结算模式在全省推广，社会保险"五险"参保达995.6万人次，城乡居民基础养老金提高9.1%，企业职工和机关事业单位退休人员基本养老金提高5.5%；低保标准提高42.4%；困难居民重特大疾病医疗救助比例提高到80%以上；残疾人康复救助标准大幅提高，0-6岁残疾儿童康复救助标准提高至3万元，提高14倍。保障性住房建设扎实推进，分配公租房10832套，建成保障房1635套。教育事业全面发展，完成新建改建扩建公办学校15所，动工建设18所，新增公益普惠性幼儿园15所，新增规范化幼儿园学位9745个，高考总录取率、本科录取率、一本录取率均居全省前列，中德合作（中山）职业技能人才培训基地建成招生，与瑞士洛桑酒店管理学院签署合作办学协议，高水平大学建设扎实推进。医疗卫生水平不断提高，综合医改全面启动，小榄医改经验获省肯定，并被中央电视台报道，家庭医生式服务重点人群签约率达65.9%，新增民营医疗机构129间。稳步推进省食品安全示范城市创建工作。更新投放公共自行车7000辆，1000 辆公交车提供免费WIFI服务。

　　（七）社会建设更加进步。第5次获评全国文明城市。深化户籍制度改革，6.7万非户籍人口落户，2.5万异地务工人员及其子女获得积分入户入学入住公租房资格。基层组织建设得到加强，277个村居委会完成换届，55名非户籍人口当选两委成员，565 人被聘为村（居）特别委员。文化建设扎实推进，大涌红木文化博览城成为国家4A 级旅游景区，孙中山故居纪念馆成为国家首批港澳青少年游学基地，建成56个基层综合性文化服务中心，举办各类文化活动6000多场次。体育事业蓬勃发展，中山籍运动员在第13届全运会上夺得金牌数和奖牌数均创历史新高，成功举办2017中山马拉松赛、中山合唱季等文体活动。"平安中山"建设不断深化，开展严打严防暴恐等专项行动，全市刑事案件发案下降5.5%，破案上升3.4%，名列中国最安全城市排行榜第5，第6次获评全国社会治安综合治理优秀地市，第4次捧回"长安杯"。社会治理体系不断完善，和谐劳动关系建设经验在全省推广，省市共建火炬开发区和谐劳动关系综合试验区启动。科学应对极端天气灾害影响，有效抵御"天鸽"等强台风侵袭。精准扶贫工作进展顺利，对口帮扶潮州市、肇庆市，对口支援西藏工布江达县、新疆生产建设兵团第三师图木舒克市、云南昭通市、四川甘孜州，以及与黑龙江佳木斯市对口合作等工作有序开展。检验检疫、安全生产、防震减灾、应急管理、民族宗教、统计、国防建设、海洋渔业、老龄、妇女儿童、红十字会、气象、人防、档案、地方志等各项工作取得新成效。

　　过去一年，我们始终注重加强政府自身建设。掀起学习贯彻党的十九大精神的热潮，用习近平新时代中国特色社会主义思想统一思想、武装头脑、指导实践、推动工作。认真履行政府党风廉政建设主体责任，加强廉政建设。推进法治政府建设，自觉接受人大依法监督、政协民主监督，认真开展建议提案办理答复工作，办复人大代表建议152件、政协提案223件，满意和基本满意率达100%。开展全市依法行政考评工作，在全省依法行政考评中获优秀等次。建立政府立法公众参与新模式。完善重大行政决策程序，落实重大行政决策合法性审查制度。聘请47位国内外知名专家担任政府顾问，所有政府组成部门与高层次智库建立起合作关系，为一大批改革发展问题提供了解决途径，促进政府决策更加科学高效。全面推进国务院行政执法"三项制度"试点工作，行政执法监督系统在全省推广。推进"数字政府"建设，在民生、政务等领域建成一批智慧项目。市行政执法信息公示平台上线运行。在全省首创政务公开法律顾问机制。推动信访工作法治化、制度化，开展律师参与化解和代理涉法涉诉信访案件试点，依法解决群众合理诉求。加强督查督办，优化政府绩效考评制度，促进行政效能提升。强化监察、审计等内部监督，推进审计监督全覆盖。严格执行中央八项规定，严格"三公"经费管理，进一步规范会议、公务用车、公务接待等公务活动。

　　各位代表！奋斗充满艰辛，成绩来之不易。回顾过去一年，大家心往一处想、劲往一处使，凝心聚力谋发展，铆足干劲抓落实，形成了百舸争流、竞相发展的生动局面。这是省委、省政府和市委正确领导的结果，是市人大及其常委会和市政协监督支持的结果，是历届政府接力耕耘、历任班子励精图治的结果，是全市干部群众团结奋斗的结果，是海内外各界人士关心帮助的结果。在此，我代表市人民政府，向全市人民，向各位人大代表、政协委员，向各民主党派、各人民团体和社会各界人士，向驻我市中央有关单位、人民解放军、武警部队官兵，向所有关心支持中山发展的港澳台同胞、海外侨胞和国内外友人，表示衷心的感谢！

　　同时，必须清醒看到，我们的工作还存在许多不足，也面临不少困难和挑战。主要是：新旧动能转换仍处于接续期，经济发展质量和效益不够高；高端创新资源紧缺，缺乏"独角兽企业""瞪羚企业"；市场秩序治理有待加强，产品质量有待提高；改革有待进一步深化，长期积累的深层次矛盾和问题有待破局；重大基础设施建设有待加强，"多规不合"、违法用地、违法建设问题仍然存在；黑臭水体、机动车尾气、背街小巷环境亟待治理，城市管理精细化水平有待提高；民生领域还有不少短板，群众对优质教育、医疗的需求还很强烈；部分公职人员担当意识不够、改革意识不强、专业化水平不高，推动发展、服务群众的能力有待提高。对这些问题，我们要切实增强忧患意识和担当意识，勇于面对、积极应对，采取果断有力措施加以解决。

■2018年工作安排

　　各位代表！2018年，是全面贯彻党的十九大精神的开局之年，党的十九大为全面迈向社会主义现代化绘就了宏伟蓝图，习近平新时代中国特色社会主义思想为我们指明了前进的方向。2018年，也是改革开放40周年，40 年来全国上下求索奋进、巨变辉煌，证明了改革开放是强国之路。2018年，还是中山成为地级市30 周年，30 年来中山人民勇立潮头、锐意进取，用实干托起了梦想。回顾过去，我们激情满怀；展望未来，我们信心百倍。综观国内外形势，当前全球经济稳定复苏，向好预期增多，我国经济保持稳中有进、稳中向好势头，将继续领跑全球经济。随着粤港澳大湾区建设上升为国家战略，随着省委、省政府赋予中山"四个定位"的深入推进，随着深中通道、深茂高铁、港珠澳大桥等重大基础设施加快建设，我市发展迎来了极为难得的重大历史机遇。同时，我市实体经济基础好，民间资本旺盛，人文底蕴深厚，生态环境良好，社会和谐稳定，在新一轮经济、文化、社会、生态等综合实力的竞争中，处于有利位置。我们要牢牢把握中山发展的历史窗口期，坚持抬头看齐、带头示范、埋头实干、回头评估、点头满意"五头并进"，以世界眼光、战略思维，对标先进城市，从中山的中山、广东的中山、湾区的中山、中国的中山、世界的中山五个维度，积极拥抱大城市、拥抱大湾区、拥抱高科技，主动谋划和推动新一轮发展，再创中山发展新辉煌。

　　2018 年政府工作的总体要求是：坚持以习近平新时代中国特色社会主义思想为指导，全面贯彻党的十九大精神，按照习近平总书记对广东工作重要指示批示要求，按照省委十二届二次、三次全会和市委十四届二次、三次全会部署，坚持稳中求进工作总基调，坚持新发展理念，贯彻高质量发展的要求，统筹推进"五位一体"总体布局和协调推进"四个全面"战略布局，积极参与推进粤港澳大湾区建设，以供给侧结构性改革为主线，以深化创新驱动为抓手，以满足人民日益增长的美好生活需要为根本，扎实做好稳增长、促改革、调结构、惠民生、防风险各项工作，加快建设创新中山、法治中山、文明中山、平安中山、美丽中山。

　　2018 年经济社会发展的主要预期目标是：地区生产总值增长7.5%左右，固定资产投资增长10%，社会消费品零售总额增长10%，一般公共预算收入增长8%，居民消费价格涨幅控制在3%以内，城乡居民收入增长与经济增长同步，城镇登记失业率控制在3%以内，主要污染物总量减排和大气、水环境质量完成省下达的目标任务。

　　做好今后一个时期工作，必须坚持"四轮驱动"工作要求：一是强化市级统筹。针对镇区办不了的事，在城乡规划、土地利用、招商引资、公共服务、环境容量等方面加强统筹，集中力量办大事，增强对中央、省、市重大决策部署的落实力、执行力。二是实施组团发展。强化规划引领，优化资源配置，以重大产业平台、重大公共基础设施建设等为有力抓手，打造一批标志性、示范性的组团项目，破解资源利用碎片化等"瓶颈"问题，推动形成强大规模效应。三是激发镇区活力。坚持重心下移，进一步简政放权，让镇区资源要素活起来，让基层干部的积极性、创造性迸发出来，形成八仙过海各显神通、劲足实干特色鲜明的基层发展格局。四是坚持分类施策。根据各组团、各镇区功能定位，精准施策，实施分类指导、分类管理，推进差异化评价考核，努力推动区域均衡协调发展。

　　为实现上述目标，必须做好以下工作。

　　一、聚焦实体经济，推动经济高质量发展

　　坚持以习近平新时代中国特色社会主义经济思想为引领，按照质量第一、效益优先的要求，把发展经济的着力点放在实体经济上，推动经济发展质量变革、效率变革、动力变革，保持经济运行在合理区间，加速向高质量发展转变。实体经济为中山发展创造了辉煌，中山走向新的辉煌也离不开实体经济。

　　加快建设制造强市。大力发展先进制造业，推动互联网、大数据、人工智能与实体经济深度融合。培育壮大人工智能产业，实施人工智能专项行动计划，筹建一批人工智能和智能制造装备技术转化中心。推动传统产业优化升级，实施新一轮技术改造行动计划，引导600家以上工业企业实施技术改造。加快重大产业平台建设，完善"园区管理中心+开发公司"管理架构，加快平台土地征收、基础设施建设、项目引进、转型升级等工作，新增建设用地优先保障产业平台开发。实施骨干企业培育工程，培育一批有特色、有竞争力、高成长性的行业龙头企业和小巨人企业，多措并举促进一批小微企业上规模、中型企业上档次。加大财政、金融对实体经济的支持力度，市财政安排产业扶持资金21.8亿元。激发和保护企业家精神，弘扬工匠精神。

　　推动服务业创新发展。完善服务业政策体系，推进国家服务业综合改革试点。大力发展新兴金融、信息服务、工业设计、文化创意、科技中介等服务业新兴业态。加大人力资源服务、现代物流、检验检测、研发设计、电子商务等生产性服务业培育力度，加快发展家庭服务、健康养老、旅游、体育等生活性服务业。实施服务业集聚区提升计划，推动服务业企业"上规上限"。加强服务业区域合作，主动承接广州、深圳、香港、澳门等地辐射。推进国家全域旅游示范区创建工作，加快重点旅游项目建设，推进"厕所革命"。

　　实施乡村振兴战略。坚持农业农村优先发展，让农民有更多获得感和幸福感。大力发展"互联网+农业"，完善生态循环农业产业链条，积极发展高科技农业，培育农产品电商、休闲农业、智慧农业等新业态。优化农业布局，推动渔业提质增效，做强做优水产、花卉苗木、水果等特色优势产业。保障农产品质量安全，完善肉菜溯源体系，启动农贸市场升级改造。完善储粮机制，保障粮食安全。深化农村集体产权制度改革，促进农村集体资产保值增值。推进农村普惠金融，撬动更多金融资源投向"三农"领域。开展农村人居环境综合整治，完成150个行政村整治，提升村庄岭南风貌特色，建设美丽乡村。促进农民创业，培育新型职业农民。加强农村基层队伍建设。

　　加大招商引资力度。完善招商工作运行机制，市属招商公司投入运营。瞄准先进装备、人工智能、健康医药、新材料、检验检测等领域，着力引进一批龙头项目。实施重点项目落地专项行动计划，健全项目落地和跟踪服务机制，强化比较优势，提供全方位保障，推动重点签约项目落地。做好现有企业培优扶强工作，促进企业增资扩产。

　　积极扩大有效投资。坚持项目带动，推动一批亿元以上项目开工建设，力促建成项目尽快投产，引导企业加强工业投资。加快推进交通、环保、水利、市政、民生等一批重点项目建设。突出抓好171个省市重点项目建设，总投资超过4000亿元，年度计划投资548.9亿元。强化重点项目亮灯管理机制，健全重点项目年度考核制度。推动政府与社会资本合作，鼓励民间资本参与基础设施和公用事业建设。

　　加强质量建设。创建全国质量强市示范城市。实施质量提升工程，提升五金产品、灯饰、淋浴房、燃气具等重点产品质量，推进出口产品质量安全示范区建设。建成灯具配件产品检测省级站，加快智能厨卫生活电器产品检验省级站建设，筹建电梯安全评估、智能家居安防产品检验等省级站，争取在新材料、新能源、机器人等领域，引进一批国际知名检验检测认证机构。强化质量监管，严厉打击假冒伪劣、虚假广告、价格欺诈等行为。加强网络消费市场监管，推广 "工商红盾电子标识"。

　　二、狠抓创新驱动，加快新旧动能转换

　　坚持把创新作为引领发展的第一动力，深入推进以科技创新为核心的全面创新，保持定力、久久为功，加快形成以创新为主要引领和支撑的经济体系，建设创新型城市。

　　增强企业自主创新能力。完善支持科技创新的政策体系，加强对企业全方位、全成长周期服务。推进高新技术企业"树标提质"行动。支持现有高新技术企业做优做强，推动更多高新技术企业进入全省百强。推动大中型企业设立研发机构，加大中小微企业研发支持力度，发展壮大一批创新型领军企业。推动产业链、创新链、资金链、政策链"四链"融合，推动金融资本、科研院所与优质高新技术企业对接。加强关键核心技术攻关，加大科技成果转化力度。

　　加快创新平台建设。深入推进珠三角国家自主创新示范区建设，积极参与国家科技产业创新中心建设。加快三角迪茵湖科创基地、坦洲科创岛建设，与火炬开发区、翠亨新区构建"两区一湖一岛"格局。发展壮大新型研发机构，加快建设一批高水平研发机构。推进孵化育成体系提质增效行动，打造"众创空间+孵化器+加速器"全孵化链条。对标全球顶尖创新资源，探索组建国际孵化器和加速器，引进清华大学、斯坦福大学、早稻田大学等一批国内外知名高校的创新团队。对接广深科技创新走廊，吸引香港、澳门科研团队落户中山。推动产学研合作和协同创新，推进技术转移和知识产权交易。

　　推动金融创新发展。支持企业上市、新三板挂牌，支持现有上市企业以各种形式投资中山。引导企业通过定向增发、发行公司债和企业债等多渠道进行直接融资。组建母基金，规范引导创业投资机构发展，鼓励股权投资基金投向本土企业。加强小微企业金融服务，大力发展普惠金融，做好转贷创新试点工作。组建金融证券研究所，推动中山农村商业银行资本运作，壮大本土金融机构体系。鼓励金融机构发展绿色金融，支持绿色产业发展。

　　加大人才培养和引进力度。实施人才优先发展战略，全面落实人才政策，对接国家、省重大人才工程，推进中山 "英才计划""优才工程"，大力培育和引进一批科技领军人物、青年科技人才、创新创业团队和紧缺适用高层次人才。加强技能人才培养。推行"中山优才卡"制度，实施人才安居工程，着力解决人才落户、住房、子女入学、医疗保障、养老、出入境等问题。支持人才创新创业，加快人才创新创业生态园建设。坚持用事业留人、平台留人、环境留人，努力让人才引得进、留得住、用得好。吸引人才很重要，留住和用好人才更重要。

　　构建全生命周期公共技术服务体系。建设从科技孵化、技术研发、产品设计、智能制造、检验检测，到品牌营销、认证服务、技能培训、法律咨询等多层次、全覆盖的服务平台体系，为企业提供全方位服务。加快推进工业设计、两化融合、软件服务、共性工厂、检验检测等新平台落地，延长平台链条。做大做强中国质量认证中心华南实验室等公共服务平台，培育一批具有全国影响力的大品牌平台。

　　营造良好创新生态。优化财政对科技创新的投入，激励企业加大研发投入。落实高新技术企业所得税减免、研发费加计扣除、技术合同登记等支持企业创新的普惠性政策。引导银行扩大科技信贷覆盖面，推进知识产权质押融资，拓宽科技型中小企业融资渠道，引导科技型企业挂牌上市。加强知识产权保护运用，推进国家知识产权示范城市建设。倡导崇尚创新、宽容失败的创新文化。

　　三、深化改革开放，激发经济社会活力

　　坚持敢为人先，树立改革强市的鲜明导向，大力弘扬"红船精神"，高举改革大旗，扛起改革担当，以改革促动破局，以开放搞活发展，用敢闯敢干、攻坚破难的劲头，投身到新一轮改革开放的大潮中去。

　　深入推进 "三去一降一补"。淘汰落后产能，化解过剩产能，为新动能发展腾出空间和资源。做好金融风险防控工作。落实国家、省减税降费政策，实施涉企收费目录清单管理，最大程度减轻企业负担。加快补齐交通、能源、环保、信息等重点领域短板，促进区域城乡协调发展。

　　加大"放管服"力度。把"放管服"改革作为优化营商环境的重要抓手，要放得更彻底、更到位，管得更科学、更高效，服务更精准、更贴心。承接好省级审批权限下放工作。深化"一门式一网式"政府服务模式改革，最大程度实现群众办事办证"马上办、网上办、一次办、代理办、同城办"。实施投资项目审批"容缺受理"机制，推进并联审批，推行项目导办代办服务，最大程度方便企业办事。深化商事制度改革，建设商事登记全程电子化智能申报审批平台。加强中介超市信息化建设，提升中介服务水平。大力推进 "数字政府"建设，构建安全高效的政务大数据平台，促进数据共享。优化镇区政务服务，推进镇区行政服务中心建设。加强社会信用体系建设，营造守信激励、失信惩戒的社会氛围。

　　推进财税制度改革。加强预算执行管理，完善市镇预算管理体制，稳步推进支出经济分类科目改革。加大转移支付力度，推进财政管理体制改革。加大公共服务领域投入，保障重要民生工程建设。发挥财政资金放大作用，引导社会资本投向重点领域和薄弱环节。加强地方政府性债务管理，防控地方债务风险，严禁违法违规融资举债。推动国地税征管体制改革，创新涉税信息共享和联合办税手段，营造良好税收秩序。

　　加快国资国企改革。完善各类国有资产管理体制，以管资本为主推动国资监管职能转变，提升国资监管能力和实效。提高国企管理水平，加快形成有效制衡的公司法人治理结构和灵活高效的市场经营机制。聚焦主业发展，壮大企业规模，做强做优做大市属国有资本。推进国有资本证券化，采取借壳、并购及自主培育等方式，推动国有企业上市上板，推进国企去杠杆、减负债。加大高层次专业人才、行业精英引进力度，培育企业家团队。

　　积极参与推进粤港澳大湾区建设。建设粤港澳大湾区世界级先进制造业基地，打造区域性综合交通枢纽、产业创新中心和历史文化名城。对标世界著名湾区，主动对接粤港澳大湾区规划，携手兄弟城市共同加快推进世界级城市群建设。以更加主动的姿态，与大湾区中心城市广州、深圳、香港互动，在规划上主动衔接，在交通上主动对接，在产业上主动承接。设立时间表和路线图，打通与周边城市间的断头路。紧密对接广东自贸区南沙、前海、横琴片区，复制推广自贸区先进经验。

　　构筑开放新格局。拓展与"一带一路"沿线支点国家、欧美发达国家交流合作。巩固提升一般贸易，发展服务贸易。培育壮大民营企业进出口力量。积极发展跨境电商，鼓励企业开展跨境电商业务。支持企业开展国际产能合作和海外并购，引导对外投资健康发展。加强口岸基础设施和核心能力建设，以完善中山国际贸易 "单一窗口"标准版为带动，推动"三互"大通关建设，进一步优化通关环境。深化国际友城交流合作，建立中山"侨谱"和海外青年侨俊交流平台，汇聚侨智侨力。

　　四、加强建设管理，提高城市功能品质

　　坚持科学规划、统筹建设，一揽子抓好规划、建设和管理，促进生产空间集约高效、生活空间宜居适度、生态空间山清水秀，让市民生活得更便捷、更舒心、更美好。

　　大力建设交通强市。加快轨道交通建设，开展城市地铁前期研究，加强与周边城市轨道网规划衔接。配合推进深茂高铁规划建设，加快南沙港铁路建设，积极推动广州、佛山地铁延伸至中山工作。加快高速公路建设，推进中开高速、东部外环高速一期、西部外环高速（含小榄支线）建设，配合推进深中通道、广中江高速、香海高速、南中高速建设。加强快线建设，完成长江路主线快速化改造，完成康华路改造，建成横栏快线 （西二环至古神公路段）、阜民路（阳光大道段）、北二环（沙港公路），推进105国道南段、民众快线、南朗快线、大涌快线等7条快线建设，动工建设古镇快线。规划建设多条过岐江河通道。加强交通微循环建设，落实《主城区近期交通改善详细规划》，开展中心城区路面升级及全要素改造。推进中心城区公共停车场建设。加快城市慢行系统建设。加强港口建设，推进"一港五区"智能化建设，提高集疏运能力。完善马鞍港区配套设施，加快新客运码头及综合体建设。加强交通管理，推进"智慧交通"建设，运用大数据、云计算、人工智能、新能源等技术，建立路、车、人之间高效信息网络，建设智慧化、共享型便捷交通服务体系。创建省级公交示范市，提高公共交通出行分担率，优化农村客运和公交线路网络，实现公交车WIFI全覆盖。加强网约车、电动车、共享单车管理。开展路域环境综合治理行动。设立交通基础设施建设基金，支持社会资本参与交通基础设施建设。

　　加强城乡规划统筹和引领。谋划编制中山总规2035，做好2049空间发展战略研究，以高质量规划引领高品质城乡发展。推进多规融合。加强总规与土规的衔接。完成城镇开发边界划定，优化城市生态控制线。建立城市设计管理制度，明确城市设计重点区域和节点。实施"强心"战略，提高中心城区首位度，加强中心城区天际线、山际线、滨水线管控。开展岐江河一河两岸城市设计，塑造滨水景观风貌。坚持开门做规划，让群众成为城市管理的主角。严格规划的刚性管控，保持城市建设不变形、不走样。

　　推进城市建设和更新。坚持新城开发建设与旧城保护改造相结合，全面改善城市总体风貌。推进组团式发展，理顺市、组团、镇区之间的关系，加快交通、体育公园、儿童活动场所等组团基础设施和公共服务设施建设，优化要素资源配置。加快岐江新城建设，逐步解决历史遗留问题，探索新时代现代化城市发展的新路子，布局一批适应大湾区世界级城市群发展的项目。推进特色小镇建设，把大涌、古镇打造成为特色小镇中的精彩范例。启动石岐总部经济区和东区总部经济区建设。推进翠亨新区科技金融中心建设，加快翠亨新区海绵型试点项目和地下综合管廊建设。提高城镇化质量，强化南部镇区和北部镇区的功能定位，建设好中山的南大门、北大门。加快电网发展，推动500千伏文山站等重点电力工程建设。启动智能电网项目建设，推进中心城区和翠亨新区电网电缆化改造。加大水利设施建设力度，提升堤围防灾抗灾水平。加强气象现代化建设，增强防灾减灾能力。加大地质灾害防治力度，整治地质灾害隐患。成片推进既有小区、城中村光纤改造。出台更加灵活有效的城市更新措施，实施"三旧"改造增减挂钩机制，重点推进连片改造项目，鼓励镇区开展微改造和旧村庄土地整备试点。推进旧楼加装电梯工作。

　　促进土地集约节约利用。开展第三次全国土地调查，摸清全市土地利用现状。加快批而未供用地清理，建立批而未供建设用地指标调整使用统筹机制。加快闲置用地处置，年内处置闲置用地不少于5000亩。推进锌铁棚改造，盘活低效工业用地和闲置厂房。严格执行耕地保护责任制，落实耕地保护经济补偿机制，建设高标准基本农田1.8万亩，开展村级土地利用规划试点。严格国土空间用途管制，加强土地卫片执法检查，大力推进违法用地、违法建设整改。将单位生产总值增长消耗新增建设用地量列入区域发展衡量指标。

　　提高城市管理水平。实施精细化管理，推行从源头到终端的全方位全过程管理。推进城市管理体制改革，提高城市管理和服务一体化水平。开展市容环境卫生综合大整治行动，加强户外广告管理。统筹规划全市移动通信基站及信息管线。建设智慧城市，让群众感受到信息化带来的便利。推广4K 电视网络应用，开通4K 电视频道。加强施工管理，最大程度降低市政工程施工对市民出行影响。

　　五、保护自然环境，建设绿色生态家园

　　坚持人与自然和谐共生，牢固树立和践行绿水青山就是金山银山的理念，落实节约资源和保护环境的基本国策，像对待生命一样对待生态环境，努力为市民创造高品质的自然环境和生态空间，建设天蓝、地绿、水清的美好家园。

　　加强环境综合治理。坚持"系统治水"思路，统筹岸上源头治理与水体综合治理，打好治水攻坚战。建立健全河道管理体系，完善河长制考核问责机制。严格执行水环境保护法律法规，将涉河活动治理纳入法治化轨道。加快推进黑臭水体整治，全面启动15个流域黑臭水体综合整治工程。做好截污纳管、控源减排、清淤活水和生态修复工作，严控工业、农业、生活污染排放。推进全流域河涌水质自动监测平台建设，加强水环境监测。坚持陆海联动，强化海洋污染防控。全面落实大气污染防治措施，建设100个空气质量微观站，突出抓好PM 2.5和臭氧污染治理，开展挥发性有机化合物综合治理，加大扬尘污染防控力度，将高污染燃料禁燃区扩展到全市域，打赢蓝天保卫战。加强土壤污染防治，做好污染地块环境监管试点工作。加快城乡环保基础设施建设，推进生活垃圾分类收集，提高生活垃圾和生活污水收集处理效率。加强农业面源污染治理，做好畜禽养殖污染整治工作。加大环保执法力度，从严从重打击环保违法行为。落实中央环保督察和国务院海洋督察反馈意见整改，解决环境突出问题。

　　加大环境保护力度。划定生态保护红线，启动饮用水源保护区生态补偿工作。加快创建国家森林城市，推进翠亨国家湿地公园、中山国家森林公园、国家级自然保护区建设，建设民众长堤、沙溪滨河、三角金鲤湾、神湾丫髻山等一批湿地公园和森林公园，推动金字山公园、儿童公园、古香林公园、金钟湖公园、气象公园、树木园（三期）等公园建设。推进全域森林小镇建设，提升城市绿化美化水平。开展全民绿色行动，构建政府、企业、社会和公众共同参与的环境治理体系。落实休禁渔制度，保护海洋与渔业生态环境。开展中心城区路面柔性化改造，减少噪音和扬尘。

　　推进节能减排降碳。严格落实约束性指标要求，确保完成省下达的节能降耗目标。强化重点用能企业节能管理，推动企业清洁生产。开展近零碳排放示范工程建设，加大碳普惠制推广力度。

　　加快推广应用新能源公共汽车，投放各类清洁能源、节能与新能源车辆，加快电源项目和充电设施建设。推动绿色科技创新，发展节能环保、清洁生产、清洁能源等产业。倡导简约适度、绿色低碳的生活方式。

　　六、加强文化建设，增强城市软实力

　　坚持文化传承创新，激发全社会文化创新创造活力，弘扬向上向善、孝老爱亲、诚信互助的社会风尚，擦亮国家历史文化名城和全国文明城市招牌，建设文化强市。

　　加强精神文明建设。积极培育和践行社会主义核心价值观，传承发展优秀传统文化，广泛开展文明村镇、文明单位、文明校园、文明家庭等群众性精神文明创建活动。举办改革开放40周年纪念系列活动，继承和弘扬中山人"敢为天下先"的改革创新精神。办好慈善万人行活动。

　　繁荣发展文化事业。实施文化惠民工程，创建国家公共文化服务体系示范区。加大公共文化服务设施建设力度，推进市图书馆、博物馆、"中山文化云"、街区自助图书馆等基础设施建设，完善图书馆和文化馆总分馆服务体系，实现村（居）基层综合性文化服务中心全覆盖。引导社会力量参与公共文化服务。坚持以人民为中心的创作导向，鼓励文艺精品创作生产。推进全民阅读活动，办好香山讲坛、书香中山、中山读书月、中山书展等品牌。举办群众合唱艺术活动，办好市民大舞台、公园音乐节、少儿艺术花会等活动。加强新型智库建设。

　　发展壮大文化产业。推进文化改革和现代文化市场建设，促进文化与科技、教育、金融、旅游等融合发展。做大做强游戏游艺、红木文化、灯饰文化等特色文化产业，培育和发展动漫设计、影视制作和数字娱乐等新兴文化产业，建设大型文化旅游综合体，打造一批文化龙头企业。加强版权保护，大力发展版权产业。

　　保护传承文化遗产。坚持在保护中发展、在发展中保护，保护是第一位的。做好杨殷故居、珠江纵队司令部等革命文化纪念地保护工作，传承红色文化。加强历史文化名镇名村、历史文化街区和城市特色风貌管理，修缮郑观应故居、张文湛故居等名人故居，修建萧友梅、吕文成展览馆，做好传统民居、历史建筑、不可移动文物的保护工作。谋划推进孙文西路、从善坊等历史文化街区的保护性开发，保留老石岐味道。推进岐澳古道活化利用。加强乡土文化建设，发掘和保护一批处处有历史、步步有文化的传统村落。制定历史底蕴清单，挖掘和保护非物质文化遗产，保护发展咸水歌、醉龙舞、飘色等传统民俗文化。成立粤剧研究院。

　　推进孙中山故里旅游景区建设。

　　完善管理机制，推进旅游区市场化专业化运作。推进旅游区停车设施、旅游休憩设施等配套基础设施建设。改造提升孙中山故居纪念馆。推进翠亨旧村保护活化，推动旅游区周边资源联动开发。

　　七、保障改善民生，增强人民群众获得感

　　坚持以人民为中心，始终把人民利益摆在至高无上的地位，大力解决群众最关心最直接最现实的利益问题，让改革发展成果更多更公平惠及全体人民，让市民获得感、幸福感、安全感更加充实、更有保障、更可持续。

　　优先发展教育事业。深化教育改革，加大教育投入，提高教育质量。实施学前教育第三期行动计划，推进学前教育扩容提质工程，增加规范化幼儿园学位6000个。加快公办中小学校建设，新建改建扩建公办学校40所。开展集团化办学改革试点，探索扩大优质教育资源的有效途径。深化中高职一体化发展，启动中职学校专业结构和布局调整，推动中职教育组团发展。促进民办教育健康发展。加强高水平大学建设，推进电子科大中山学院、广东药科大学省市共建本科院校发展，加快与德国、瑞士中外合作办学项目建设。实施新一轮强师工程，加强师德师风建设，培养高素质教师队伍。落实立德树人根本任务，强化学校德育工作和高校思想政治工作。推进智慧校园建设，提高教育信息化水平。

　　加强社会保障体系建设。实施全民参保登记计划，推进社会保险全覆盖。落实城镇职工基本养老金和城乡居民基础养老金正常调整机制，配合实施养老保险省级统筹。落实失业保险稳岗补贴政策。完善 "互联网+社保"，优化社会保险管理服务。完善社会救助体系，提升重特大疾病医疗救助水平，做好慈善救助，加强残疾人服务保障。坚持房子是用来住的、不是用来炒的定位，严格落实国家房地产市场调控政策，严厉打击房地产企业和中介机构违法违规行为，坚持租购并举，完善多主体供应、多渠道保障的住房制度。

　　提升就业创业质量。坚持就业是最大的民生，实施就业优先战略和积极就业政策，推动更高质量和更充分的就业创业。完善就业创业扶持政策，提供全方位公共就业服务。加强劳动者技能培训。做好大学生、残疾人、就业困难人员等重点群体就业创业工作，确保家庭困难毕业生100%就业。开展专项用工服务，帮助企业解决招工难题。

　　推进健康中山建设。加强医联体建设，推广小榄医改经验，完善分级诊疗、双向转诊保障机制。健全现代医院管理制度，在架构、权限、职责等方面与国内先进医院接轨。探索建设区域性医疗中心，引进国内外顶尖医疗专家团队。加强卫生信息化建设，方便群众就医。完善药品供应保障制度，解决药价虚高问题。推进市人民医院新院区、市儿童医院建设，加快市第三人民医院改扩建。加强全科医生培养。提高家庭医生式服务重点人群签约率，提高签约服务质量。大力发展和规范社会办医。加大疾病综合防控力度，提高卫生应急能力。完善全面两孩配套政策，实施儿童体质健康提升行动，提高妇幼健康服务水平，全面推进公共场所母婴室建设。推进计生服务管理改革，加强计生家庭扶助。创建省食品安全示范城市，实行最严格的食品药品安全监管制度，保障群众饮食用药安全。广泛开展全民健身活动，建设更多市民身边的公共活动场所。培育发展体育产业和体育市场。

　　深入推进平安中山建设。整合优化警力资源配置，提高快速反应能力。推进"忠诚走前列、护航新征程"粤警行动计划，围绕平安建设重点难点问题，持续开展严打严防暴恐专项行动，严厉打击黑拐枪、盗抢骗、黄赌毒等突出违法犯罪行为。加快"智慧公安"重点项目建设，推进警务大数据平台、云计算、智能感知、视频专网等信息化建设。完善立体化、信息化社会治安防控体系，实现 "公安+辅警+群防群治"三级联动，提升社会治安管理社会化、智能化、专业化水平。加快反恐指挥中心建设，推进全市110统一接处警工作，实现"图情指行"一体化和"一点发动、全城响应"。加强出租屋安全管理。加强安全生产综合治理，做好消防工作，坚决防范和遏制重特大事故发生。落实国家安全观，推进国家安全人民防线建设。

　　加强和创新社会治理。发挥律师、心理咨询师、社工等专业人士作用，推动全民参与社会治理。优化流动人口激励机制，完善积分制，打造"流管政务服务24小时网上超市"。加大全民普法力度，完善公共法律服务体系，实现刑事案件律师辩护全覆盖。做好综治维稳工作，加强社会风险评估和矛盾纠纷排查化解，完善信访、人民调解、仲裁、诉讼等多元化矛盾纠纷解决机制，建设和谐劳动关系。

　　办好十件民生实事。从群众的实际需求出发，继续集中力量办好十件民生实事。一是优化医疗服务；二是推进交通畅行；三是改善水体质量；四是缓解停车紧张；五是扩充教育资源；六是关爱老年人群；七是完善公交设施；八是帮扶大病群体；九是保障食品安全；十是开放一批公园。

　　积极发展各项社会事业。做好海外侨胞和归侨侨眷服务工作，让海外乡亲感受到家乡温暖。坚持扶贫同扶志、扶智相结合，做好对口扶贫和支援工作。加强国防建设，做好做优双拥工作，加强复退军人服务保障。贯彻落实《中长期青年发展规划（2016-2025）》。做好检验检疫、台务、民族宗教、妇女儿童、统计、档案、地方志等工作。

　　八、加强自身建设，打造法治廉洁高效政府

　　坚持和加强党的全面领导，切实增强"四个意识"，坚决维护党中央权威和集中统一领导，自觉在思想上政治上行动上同以习近平同志为核心的党中央保持高度一致，严守政治纪律和政治规矩，全面推进政治建设、思想建设、组织建设、作风建设、纪律建设，把制度建设贯穿其中，通过政府自身改革和建设，提升治理体系和治理能力的现代化水平。

　　深入开展党的十九大精神学习贯彻工作。将学习贯彻党的十九大精神作为首要政治任务，认真开展"不忘初心、牢记使命"主题教育。围绕学懂弄通做实，开展"大学习、深调研、真落实"活动，推动政府系统学习贯彻往深里走、往实里抓。围绕党的十九大提出的新思想、新目标、新战略、新举措，完善政府工作思路，细化工作举措，让习近平新时代中国特色社会主义思想在中山落地生根、结出丰硕成果。

　　坚持推进依法行政。深入贯彻全面依法治国要求，建设法治政府。依法接受人大及其常委会的监督，自觉接受政协的民主监督，主动接受社会和舆论监督。深入推进重点领域政府立法，提高政府立法公众参与度。建立政府立法工作事项第三方评估制度。完善行政争议诉前联调机制。严格规范公正文明执法，改革完善城管、环保执法体制机制，强化用地、环保等重点领域的行政执法。实施"七五"普法规划，深入开展法治宣传和法治创建活动。深化政务公开，加强信息发布和政策解读，及时回应社会关切，打造阳光政府。

　　持续推进正风肃纪。落实全面从严治党要求，把党风廉政建设和反腐败工作不断引向深入。坚持把纪律和规矩挺在前面，并贯穿于政府工作各个方面，持之以恒推动中央八项规定精神落实落细。大力整治表态多调门高、行动少落实差等形式主义、官僚主义突出问题，坚决防止"四风"反弹回潮。大力整治文山会海，继续压减市政府文件和会议数量。严格财经纪律，加强"三公"经费管理，加大审计改革力度，完善行政部门内控机制。持续推进反腐倡廉，保持惩治腐败的高压态势，坚持无禁区、全覆盖、零容忍，铲除侵犯群众切身利益的"微腐败"，坚决惩治侵害群众利益的不正之风。

　　积极营造干事创业氛围。发扬钉钉子精神，坚持目标导向、问题导向和结果导向，以永不懈怠的精神状态和一往无前的奋斗姿态，主动作为、动真碰硬，以实干推动发展。强化督查督办和追责问责，铆紧各方责任，层层传导压力，严厉整肃庸政懒政怠政行为，不折不扣地落实好中央、省和市委各项决策部署。深化政府绩效管理，健全激励机制和容错纠错机制，给干事者鼓劲、为担当者撑腰。把雷厉风行与久久为功结合起来，倾力营造敢担当、善作为、风清气正的干事创业氛围。

　　各位代表！蓝图已经绘就，实干铸就辉煌。让我们紧密团结在以习近平同志为核心的党中央周围，在省委、省政府和市委的坚强领导下，不忘初心、牢记使命，抢抓机遇、干在实处，奋力开创新时代中山改革发展新局面！
